# Mesenchymal Stem Cell Induced Foxp3(+) Tregs Suppress Effector T Cells and Protect against Retinal Ischemic Injury

**DOI:** 10.3390/cells10113006

**Published:** 2021-11-04

**Authors:** Mona Agrawal, Pratheepa Kumari Rasiah, Amandeep Bajwa, Johnson Rajasingh, Rajashekhar Gangaraju

**Affiliations:** 1 Department of Ophthalmology, University of Tennessee Health Science Center, Memphis, TN 38163, USA; magrawa1@uthsc.edu (M.A.); prasiah@uthsc.edu (P.K.R.); 2 James D. Eason Transplant Institute, Department of Surgery, University of Tennessee Health Science Center, Memphis, TN 38163, USA; abajwa@uthsc.edu; 3 Department of Genetics, Genomics, and Informatics, University of Tennessee Health Science Center, Memphis, TN 38163, USA; 4 Department of Microbiology, Immunology, and Biochemistry, University of Tennessee Health Science Center, Memphis, TN 38163, USA; rjohn186@uthsc.edu; 5 Department of Bioscience Research, University of Tennessee Health Science Center, Memphis, TN 38163, USA; 6 Department of Anatomy & Neurobiology, University of Tennessee Health Science Center, Memphis, TN 38163, USA

**Keywords:** CD4+CD25+, retinopathy, inflammation, iPSC, mitochondria

## Abstract

Mesenchymal stem/stromal cells (MSC) are well known for immunomodulation; however, the mechanisms involved in their benefits in the ischemic retina are unknown. This study tested the hypothesis that MSC induces upregulation of transcription factor forkhead box protein P3 (Foxp3) in T cells to elicit immune modulation, and thus, protect against retinal damage. Induced MSCs (iMSCs) were generated by differentiating the induced pluripotent stem cells (iPSC) derived from urinary epithelial cells through a noninsertional reprogramming approach. In in-vitro cultures, iMSC transferred mitochondria to immune cells via F-actin nanotubes significantly increased oxygen consumption rate (OCR) for basal respiration and ATP production, suppressed effector T cells, and promoted differentiation of CD4+CD25+ T regulatory cells (Tregs) in coculture with mouse splenocytes. In in-vivo studies, iMSCs transplanted in ischemia-reperfusion (I/R) injured eye significantly increased Foxp3+ Tregs in the retina compared to that of saline-injected I/R eyes. Furthermore, iMSC injected I/R eyes significantly decreased retinal inflammation as evidenced by reduced gene expression of *IL1β*, *VCAM1*, *LAMA5*, and *CCL2* and improved b-wave amplitudes compared to that of saline-injected I/R eyes. Our study demonstrates that iMSCs can transfer mitochondria to immune cells to suppress the effector T cell population. Additionally, our current data indicate that iMSC can enhance differentiation of T cells into Foxp3 Tregs in vitro and therapeutically improve the retina’s immune function by upregulation of Tregs to decrease inflammation and reduce I/R injury-induced retinal degeneration in vivo.

## 1. Introduction

Ischemic retinopathies, including diabetic retinopathy (DR), retinopathy of prematurity (ROP), and retinal vascular occlusion (RVO), are increasing in prevalence, represent a significant economic burden, and are major causes of vision loss and blindness worldwide [1,2,3]. A wide variety of traditional treatment therapies, including photocoagulation and anti-VEGF therapies during the neovascularization phase, showed benefits, with no treatments currently approved that address underlying proinflammatory pathways that are known to trigger neurovascular degeneration [2]. Stem cell therapies, mainly multipotent mesenchymal stem cells (MSCs), recently gained significant attention as a potential therapy for the treatment of ischemic retinopathies [3,4]. Our previous studies utilizing MSCs derived from the stromal vascular fraction of adult human adipose tissue (adipose-derived stem cells, ASC) [5,6], and independently corroborated with bone marrow (BM-MSC) [7] and umbilical cord (UC-MSC) [8], demonstrated substantial regeneration and recovery of the damaged retina after treatment, although the exact mechanisms by which MSCs may protect against vision loss, remains unclear.

MSCs are well known for being involved in immunomodulation, in part by donating mitochondria to damaged tissue or cells [9,10,11]. Recent evidence also suggests that MSCs can modulate T regulatory cells (Tregs) [12,13], in particular, Tregs expressing the Forkhead box P3 (Foxp3) transcription factor, which is part of the adaptive immune system and are principal regulators of inflammation and immune homeostasis [14]. The Tregs can migrate to the diseased tissue and dampen inflammation by increasing the milieu of anti-inflammatory cytokines and activating macrophages to clear the debris and restore the damaged tissue [15,16,17]. Since CD4+ T cells may mediate retinal ganglion cell (RGC) degeneration and loss of retinal function after injury [18], it may be possible to reprogram the CD4+ T cells at the site of injury to acquire a Treg phenotype. This might aid in the rescue of retinal damage and, as a result, decrease cell loss and enhance immunotolerance [19]. Therefore, in the present study, we hypothesized that intravitreal injection of MSCs in ischemia-reperfusion (I/R) injured retina reprogram CD4+ T cells to Tregs, dampen inflammation, and improve visual function. Interestingly, our data visualized that MSC actively transferred mitochondria to immune cells to suppress effector T cells and promoted differentiation of CD4+CD25+ Tregs in coculture with mouse splenocytes. Intravitreal injection of MSCs in the I/R eye significantly increased Tregs in the retina, decreased retinal inflammation, and improved visual function compared to saline-injected I/R eyes. These findings indicate that harnessing the immunosuppressive capacity of MSCs is a potential therapy for the treatment of ischemic retinal diseases.

## 2. Materials and Methods

### 2.1. Cell Isolation and Culture

iMSCs were prepared from human urinary tubular epithelial cells (UEs) through the generation of iPSCs via reprogramming with a cocktail of Oct-4, Sox-2, Klf-4, c-Myc, and Lin-28 mRNAs and subsequently differentiated into MSC as described by us previously [20,21]. Briefly, iMSCs from healthy human urine tubular epithelial cells (UEs) expressing epithelial markers CK19 and ZO1 were used to generate iPSCs via reprogramming with a cocktail of Oct-4, Sox-2, Klf-4, c-Myc and Lin-28 mRNAs [20]. To induce MSC differentiation, the UE-iPSCs were cultured under conditions conducive for mesenchymal differentiation in Mesencult ACF plus medium for 18–21 days and were characterized for MSC markers. iMSC were positive for CD105, CD90, and CD73 while negative for CD31 and CD45 (Supplemental Figure S1). To validate the mechanism of action of iMSC, ASC was used as a well-known control in in-vitro studies. ASCs used in the current study were obtained from Lonza (Cat#PT-5006), cultured in EGM-2MV media, and used between p2 and p7 in all experiments as previously described [22]. All studies involving human ASC and iMSC were approved for research as per the University of Tennessee Institutional Biosafety and as an exempt study by the Institutional Review Board. Human monocytes, THP-1 cells were purchased from ATCC (Cat#TIB-202) and cultured in RPMI 1640 complete medium as a suspension culture. In addition, primary mouse splenocytes were prepared as described previously [23]. Briefly, 6–8 weeks old wild type (C57BL/6) mice were euthanized, the spleen was collected and washed in PBS. The tissue was crushed on a 70-micron cell strainer with 5 mL syringe plunger, rinsed with RPMI 1640 media; cells were pelleted by centrifugation. Subsequently, RBCs were lysed using ACK lysis buffer, inactivated the reaction using 10% FBS. Finally, splenocytes were pelleted and resuspended in RPMI 1640 complete media and filtered through 40-micron cell strainer. Trypan blue negative splenocytes were counted as live cells and used for experiments. All cell cultures were maintained at 37 °C and 5% CO_2_ in a humidified atmosphere.

### 2.2. Coculture and Microscopy

iMSC or ASC were plated at 1 × 10^6^ cells/cm^2^ in a 60 mm dish and we let them adhere. Following this, 1 × 10^6^ cells were stained with fluorescent MitoTracker Red CMXRos (100 nm, Life Technologies, Grand Island, NE, USA) for 45 min at 37 °C. Labeled cells were washed 2× with PBS, trypsinized, and seeded into a 6-well plate containing 10 mm coverslips at 1 × 10^5^ cells per well. On the second day, 1 × 10^6^ THP-1 cells labeled with CellTracker fluorescent probe green (0.5 µM, Life Technologies) were cocultured with iMSC or ASC in a 1:10 ratio for 24 h. After 24 h of coculture, cells were fixed with 4% paraformaldehyde (PFA) and stained with DAPI nuclear stain, mounted using ProLongTM diamond antifade mountant (Life Technologies). Similarly, 1 × 10^6^ iMSC or ASC were stained with fluorescent MitoTracker Green (100 nm, Life Technologies) to assess the mitochondrial transfer through F-actin nanotubes. Following this, 1 × 10^5^ cells were seeded into a 6 well plate containing 10 mm coverslips. On the second day, 1 × 10^6^ THP-1 cells labeled with CellTracker CMAC blue (0.5 µM, Life Technologies) were cocultured with iMSC or ASC in 1:10 ratio for 24 h and treated with 350 nm of cytochalasin B (CytoB, Sigma-Aldrich, Inc., St. Louis, MO, USA). Tubular microstructure tunneling nanotubes were assessed by costaining for F-actin using Phalloidin- Tetramethylrhodamine B isothiocyanate (Sigma) in the presence or absence of CytoB. The mitochondrial transfer was assessed from images captured with an EVOS fluorescence microscope (Life Technologies) or captured with a laser scanning confocal microscope (Zeiss LSM 710, Carl Zeiss Microscopy, LLC, White Plains, NY, USA). For quantification of mitochondrial transfer, THP-1 positive for CMAC blue in the coculture experiments were identified with a region of interest (ROI) and the pixel intensities of MitoTracker Green were computed for each ROI using ImageJ. At least 20 cells/image were considered, and the data were expressed as Mean fluorescent intensity (MFI) values/cell.

### 2.3. Seahorse Flux BioanalyzerV

THP-1 cells were pretreated for 2 h with 500 nM Rotenone (acts as a potent inhibitor of complex I of the mitochondrial respiratory chain). Following this, cells were washed (2×) and cocultured with iMSC or cultured as a monoculture. Cocultured THP-1 cells were transferred to a Seahorse 24-well tissue culture plates and oxygen consumption rate (OCR) was measured, and parameters were calculated as previously described [24]. Briefly, prior to the assay, the media was changed to unbuffered DMEM (Gibco #12800-017, pH 7.4, 37 °C), and cells were equilibrated for 1 h at 37 °C. After measuring basal respiratory rate, Oligomycin (Sigma; 1 μM; uncouples ATP-coupled respiration by inhibiting ATP synthase), FCCP (Sigma; 1 μM; carbonyl cyanide 4-(trifluoromethoxy)-phenylhydrazone (FCCP), mitochondrial uncoupling agent; uncouples mitochondrial respiration from ATP to determine maximal respiratory rate), and electron transport chain (complex I and III) inhibitors, rotenone (Sigma; 0.5 μM) and antimycin A (Sigma; 0.5 μM; to eliminate all mitochondrial respiration) were injected sequentially during the assay. Basal mitochondrial respiration and ATP-linked respiration were determined in whole cells.

### 2.4. Coculture and Flow Cytometry

About 1 × 10^5^ MitoTracker green-labeled iMSC or ASC seeded in 12-well cell culture plate for 24 h. The next day, 1 × 10^6^ freshly prepared splenocytes (1:10 ratio as described above) were added to the top of the stem cell monolayer in complete RPMI 1640 media. After 24 h of coculture, non-adherent and loosely bound splenocytes were collected, washed 2× with PBS. Single-cell suspension of splenocytes was stained in FACS buffer (2% FBS in PBS), incubated with the Fc-block anti-CD16/CD32 (2.4G2) antibody followed by a panel of fluorochrome-coupled antibodies (Table 1). While viability dye eFluor 506 (eBioscience, San Diego, CA, USA) excluded dead cells, a MitoTracker dye uptake assessed mitochondrial transfer by flow cytometry. Stem cells were pretreated for 2 h with 500 nM Rotenone to study the functional relevance of mitochondria transfer and cocultured with primary mouse splenocytes.

To assess Treg differentiation, freshly prepared splenocytes were activated in the presence of coated anti-CD3 (BD, 10 µg/mL) and soluble anti-CD28 antibody (BD, 1 µg/mL) and recombinant IL-2 (PeproTech, Cranbury, NJ, USA, 1 ng/mL) for 24 h. Activated splenocytes were cocultured on the monolayer of iMSC or ASC in differentiation medium [RPMI 1640 medium, 10% FBS, 1 ng/mL IL-2 (PeproTech) and 5 ng/mL TGF-β (R&D)] for five days with media change at day 3. After 5 days of coculture, nonadherent and loosely bound splenocytes were collected, washed 2× with PBS. Single-cell suspension of splenocytes was then analyzed by flow cytometry as described above using specific surface markers (Table 1). For Foxp3 intracellular staining, cells were fixed and permeabilized using Foxp3/Transcription Factor Staining Buffer (eBioscience). Following this, cells were incubated with anti-Foxp3 (eBioscience) antibody for 50 min, washed and resuspended in the FACS buffer, kept at 4 °C, protected from light till acquisition. Data were acquired using a Bio-Rad ZE5 cell analyzer and analyzed by FlowJo software v10.8 (Flowo, Ashland, Wilmington, DE, USA).

### 2.5. RNA Isolation and Quantitative RT–PCR

Total RNA was isolated from cells using Nucleospin miRNA isolation kit (Macherey-Nagel Inc, Allentown, PA, USA), following the manufacturer’s protocol. RNA quality and integrity were measured by absorbance at 260/280 and 260/230 nm using NanoDrop 1000 spectrophotometer (Thermo Fisher Scientific, Waltham, MA, USA). cDNA was prepared in a reverse transcription reaction using 500 ng of RNA and a high-capacity cDNA reverse transcription kit (Thermo Fisher Scientific) following the manufacturer’s instructions. About 100 nanograms of template RNA and 5 nM of each forward and reverse human-specific primers related to nanotube formation (*ACTN1*, *NEXEN*, *CAP2*, and *18S*) (Table 2) were used in SYBR green-based qPCR. Total RNA was isolated from Sham, I/R injured, and I/R injured with iMSC treated mice retina at day 7 postinjury, and gene expression was quantified using TaqMan probe-based gene-specific mouse primers (Table 3) and accompanying Master Mix (Applied Biosystems, Foster City, USA) using QuantStudio 3 (Applied Biosystems) Real-Time PCR system. Data were expressed as relative gene expression or fold mRNA expression using the 2^−ΔΔCt^ method and normalized to *18S* housekeeping gene.

### 2.6. Mice, Retinal IR Injury, Electroretinography, and Immunohistology

Animal studies were approved by the Institutional Animal Care and Use Committee, University of Tennessee Health Sciences Center (UTHSC), Memphis (IACUC ID: 20-0152, Approved 16 June 2020) following the guidelines as per the Association for Research in Vision and Ophthalmology (ARVO) Statement for the Use of Animals in Ophthalmic and Vision Research. C57BL/6 wild-type (B6) mice, between 12- and 16-weeks-old, were used for the study. Animals were housed under a 12-h light/dark cycle and kept under pathogen-free conditions with unlimited food and water supply. Mice were anesthetized with a mixture of 70–90 mg/kg of ketamine and 0.04–0.08 mg/kg of dexdomitor (Orion Pharma Animal Health, FI-02101 Espoo, Finland). Retinal I/R injury was induced unilaterally in the right eye. The pupil was dilated with 1% tropicamide (Akorn, Inc., Lake Forest, CA, USA), and 0.5% proparacaine hydrochloride (Alcon Laboratories, Inc., Fort Worth, TX, USA) was applied topically onto the cornea. The eye’s anterior chamber was cannulated under microscopic guidance with a 32 1/2 inch-gauge needle connected to a silicone infusion line providing a balanced salt solution (Baxter, Deerfield, IL, USA), avoiding injury to the corneal endothelium, iris, and lens. Retinal ischemia was induced by raising intraocular pressure of cannulated eyes to 70 mm Hg (as measured by iCare Tonovet) for 60 min by elevating the saline reservoir. Whitening of the fundus was observed to ensure the induction of retinal ischemia. After 24 h of injury, about 1000 iMSCs/2 µL saline were intravitreally injected into the IR injured eye. Following this, on day 7, ERG (Celeris Rodent Electrophysiology system, Diagnosys LLC, Lowell, MA, USA) was recorded as described in previous publications [25]. Briefly, animals were dark-adapted overnight and anesthetized with ketamine (50 mg/kg) and dexmedetomidine (0.25 mg/kg) cocktail. Pupil dilation was achieved with 1% tropicamide. The electrodes were positioned on the surface of both the corneas. Light pulses were delivered at a frequency at 0.01, 0.1, and 1 cd-s/m2, and the responses were recorded simultaneously from both eyes. All the offline analyses were done with Diagnosys software to calculate b-wave amplitudes. At least three-five responses to light stimuli were averaged to determine the b-wave amplitude. Following ERG, mice were euthanized, enucleated the eye, fixed in paraformaldehyde; the retinal cup was isolated. Retinas were permeabilized and blocked before incubation with the primary antibody. Retinas were immuno-stained with anti-Foxp3 antibody (1:200, Cell signaling) for 48 h followed by incubation with goat antimouse IgG Alexa Fluor 546 secondary antibody. To distinguish vasculature, retinas were incubated with Alexa Fluor 488-Isolectin B (Invitrogen, Carlsbad, USA) and flat-mounted on a glass slide using ProLongTM diamond antifade mountant (Life Technologies). Imaging of retinal flat mounts was examined under a laser scanning confocal microscope (Zeiss LSM 710). The number of Foxp3 positive cells were counted in a blinded fashion from each image from all groups, and results were expressed as Foxp3 positive cells per square millimeter of the retina.

### 2.7. Statistical Analysis

Results are expressed as mean ± SEM for all experiments. One-way ANOVA followed by post hoc *t*-tests with the Bonferroni correction was used for multiple group comparisons using GraphPad Prism software 6.0. Comparisons of ERG data between the groups were performed using the Student’s *t*-test. Values of *p* < 0.05 were considered statistically significant.

## 3. Results

### 3.1. Mitochondrial Transfer from MSCs to Immune Cells

**Figure 1:**
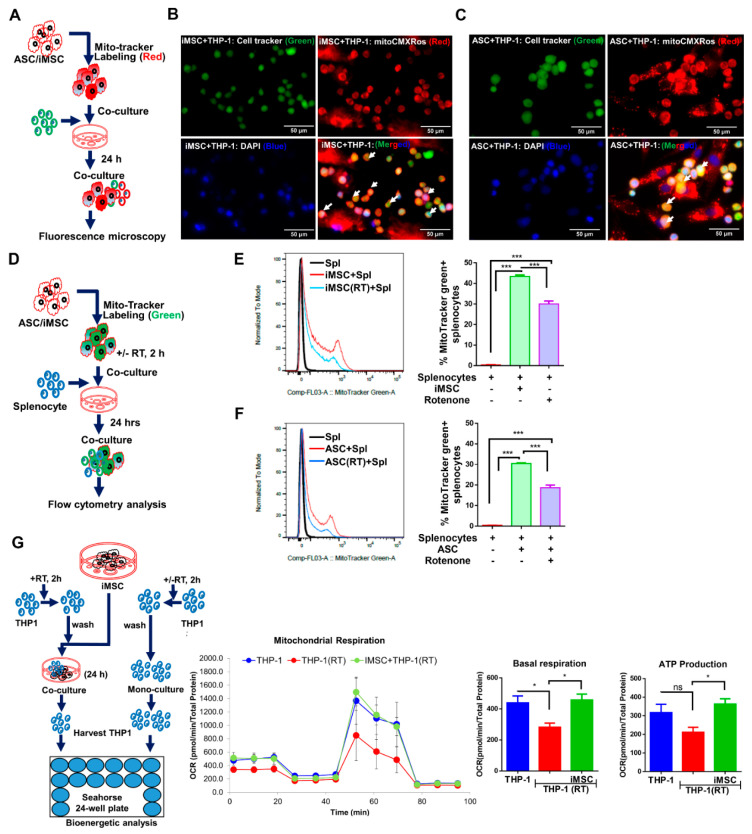
Both iMSC and ASC effectively transfer mitochondria to immune cells. (**A**) Schematic representation of stem cells and THP-1 coculture setup and fluorescence microscopy analysis. (**B**) MitoTracker CMXRos labeled iMSC (Red) or (**C**) ASC (Red) independently cocultured with cell tracker green-labeled THP-1 cells (Green) for 24 h, stained with nuclear dye DAPI (Blue), and images captured under fluorescence microscope shows increased donor-derived mitochondria in THP-1 cells (yellow; arrows). (**D**) Schematic representation of stem cells and THP-1 coculture setup and flow cytometry analysis. (**E**) Overlays and histograms showing flow cytometry analysis of mitochondria transfer from iMSC and (**F**) ASC to mouse splenocytes cocultured at 1:10 ratio. MitoTracker green-labeled iMSC and ASC pretreated with rotenone (RT) (500 nm, 2 h) and without rotenone treatment cocultured with splenocytes for 24 h. Mitochondria transfer to splenocytes is expressed as percent MitoTracker positive green splenocytes. Flow cytometry data were analyzed using Flowjo (v10.8) software and represented as mean ± SEM from triplicates of same experiment and statistical analysis by one-way ANOVA with Bonferroni correction (* *p* < 0.01, *** *p* < 0.0001, ns-not significant). (**G**) Schematic representation of stem cells and THP-1 coculture setup and OCR analysis. iMSC and RT treated THP-1 coculture was set up at 1:1 ratio for 24 h. From coculture, THP-1 were harvested and seeded onto a Cell-Tak coated Seahorse XF-24e V7 PS cell culture microplate, and oxygen consumption rate (OCR) was determined, followed by quantification of basal OCR and ATP production. Data shown as mean ± SEM from a single experiment repeated independently with similar results (* *p* < 0.01, *** *p* < 0.0001 one-way ANOVA).

To explore whether MSC can transfer their mitochondria to the immune cells, donor cells (iMSC or ASC) labeled with mitochondria-specific fluorescent probes (CMXRos red) were cocultured with CellTracker (green) labeled THP-1 cells in 1:10 ratio for 24 h (Figure 1A). Fluorescence imaging of cocultures revealed both iMSC and ASC can successfully transfer their mitochondria to recipient (THP-1) cells, as shown by colocalization of red fluorescent mitochondria in green labeled THP-1 cells (yellow, Figure 1B). To study the functional relevance of mitochondria transfer, rotenone challenged MSC were cocultured with primary mouse splenocytes at a 1:10 ratio. Mitochondria-specific fluorescent probe (MitoTracker green) labeled iMSC or ASC were treated with rotenone (500 nm, 2 h) and without, cocultured with primary mouse splenocytes, and analyzed by flow cytometry after 24 h of coculture (Figure 1C). Flow cytometry analysis demonstrated mitochondria transfer from stem cells to splenocytes (Figure 1D). While splenocytes cocultured with native iMSC had 43.4 ± 0.38 percent of MitoTracker green positive splenocytes, rotenone treated cocultures showed only 30 ± 0.81 percent positive cells (Figure 1D; *p* < 0.001). Similarly, native and rotenone treated ASC when cocultured with splenocytes demonstrated 30.4 ± 0.3 and 18.67 ± 1.3 (Figure 1E; *p* < 0.001), percent of MitoTracker green positive splenocytes, respectively. Incubation of primary mouse splenocytes with culture medium obtained by MitoTracker-labeled ASCs, but without ASCs, failed to demonstrate any fluorescence signal in splenocytes (Supplemental Figure S2), ruling out the possibility of passive transfer of the mitochondrial stain to splenocytes due to MitoTracker probe leak. To determine if the changes in mitochondrial content also altered mitochondrial function, bioenergetic analysis was undertaken. THP-1 cells pre-incubated with rotenone significantly blunted oxygen consumption compared to untreated cells as expected (Figure 1F, blue to red line). Upon coculture with iMSC, those cells exposed to rotenone demonstrated near-normal basal respiration. When ATP production was quantified, rotenone treated THP-1 cells showed a reduced ATP production though the data did not reach statistical significance while those cells cocultured with iMSC demonstrated significantly greater ATP production, which is not significantly different from untreated THP-1 cells (Figure 1F; *p* < 0.05).

### 3.2. MSC Efficiently Transfer Mitochondria in a Dose-Dependent Manner to CD4+ and CD8+ T Cells

**Figure 2:**
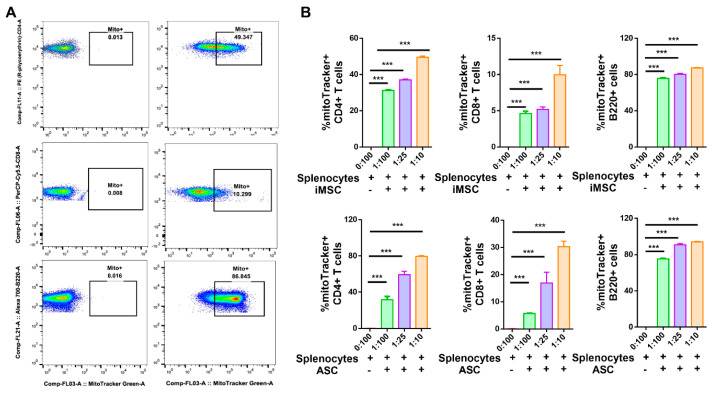
Dose-dependent transfer of mitochondria from iMSC or ASC to mouse T and B cells *in vitro*. (**A**) Representative flow cytometry dot plots showing increased MitoTracker positive CD4+ T, CD8+ T, and B220+ cells in splenocyte-iMSC coculture compared to that of monoculture. (**B**) Quantification of percent mitochondria transfer to CD4+ T, CD8+ T, and B220+ B cells increased with decreased ratio with both iMSC (upper panel) and ASC (lower panel). Data shown as mean ± SEM from a single experiment repeated independently with similar results (*** *p* < 0.0001 one-way ANOVA).

To further explore the mitochondrial transfer to specific cell populations in splenocytes, mitochondria-specific fluorescent probes (MitoTracker green) labeled iMSC or ASC were cocultured with primary mouse splenocytes at different ratios and analyzed by flow cytometry. After 24 h of coculture, nonadherent splenocytes analyzed demonstrated mitochondria transfer from stem cells into all studied lymphocyte subsets, mainly directed to B220+ B lymphocytes (86.8%), T helper CD4+ (50.68%) rather than T cytotoxic CD8+ (10.29%) lymphocytes (Figure 2A and Supplemental Figure S3; *p* < 0.001). Interestingly, donor iMSCs increased mitochondria transfer to T cells (CD4+ and CD8+) with splenocytes at increasing ratios (1:100, 1:25, and 1:10), with an average number of mitochondria of 31.2, 37, and 49.4% respectively in CD4+ T cells and with an average number of mitochondria of 4.6, 5.1 and 9.9% respectively for in CD8+ T cells (Figure 2B; *p* < 0.001). Similarly, when donor ASCs were cocultured with recipient splenocytes at increasing ratios (1:100, 1:25, and 1:10), an average of 31.7, 59.2, and 79.5% cells demonstrated mitochondria in CD4+ T cells and with an average of 5.7, 16.9, and 30.3% for mitochondria in CD8+ T cells, respectively (Figure 2B; *p* < 0.001). The level of mitochondria transferred to B220+ B cells from iMSCs or ASCs cocultured with primary mouse splenocytes ranged from 78–85% with minimal change noted with increasing coculture ratio (Figure 2B; *p* < 0.001). Altogether, these results indicate that both iMSCs and ASCs can transfer their mitochondria efficiently into primary mouse splenocytes.

### 3.3. Tunneling Nanotubes Mediate Mitochondrial Transfer from MSC to THP-1 Cells

**Figure 3:**
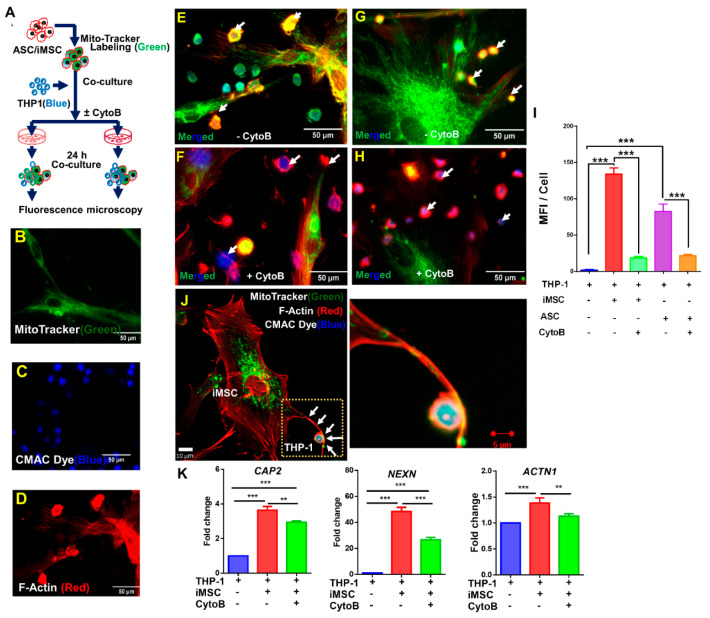
Tunneling nanotubes mediate mitochondrial transfer from iMSC to THP-1 cells. (**A**) Schematic representation of experimental design of iMSC or ASC coculture with THP-1 cells in presence and absence of cytochalasin-B. (**B**–**H**) Representative fluorescence microscopy images showing MitoTracker green positive iMSC (**B**), CMAC blue positive THP-1 cells (**C**), F-Actin red positive ASC (**D**), ASC and THP-1 coculture in absence (**E**) and in presence (**F**) of cytochalasin-B and, iMSC and THP-1 coculture in absence (**G**) and presence (**H**) of cytochalasin-B. (**I**) Mitochondria transfer from iMSC or ASC to THP-1 cells reduced with cytochalasin-B. Mean fluorescent intensity (MFI) values calculated using ImageJ. Data shown as mean ± SEM from a single experiment (*** *p* < 0.001 one-way ANOVA). (**J**) Representative Confocal images of intercellular mitochondrial transfer between iMSC and THP-1 via F-Actin positive nanotubes. Marked area magnified to show MitoTracker positive mitochondria in F-actin positive nanotube. (**K**) qRT-PCR analysis of genes related to nanotubes formation (CAP2, NEXN, and ACTN1) increased significantly during mitochondria transfer from donor (iMSC) to recipient (THP-1) cells. On other hand, cells exposed to cytochalasin-B significantly reduced CAP2, NEXN, and ACTN1 expression. Data shown as mean ± SEM from a single experiment repeated independently with similar results (** *p* < 0.001, *** *p* < 0.0001 one-way ANOVA).

After confirming the mitochondrial transfer from iMSCs or ASCs to immune cells next, we investigated whether F-actin-positive tubular microstructure known as tunneling nanotubes (TNTs) are involved in intercellular mitochondrial transfer from donor to recipient cells. To this end, MitoTracker green-labeled iMSCs or ASCs were cocultured with CMAC blue labeled THP-1 cells with and without CytoB (Figure 3A–D). As expected, fluorescence imaging revealed the transfer of mitochondria (green) from iMSC or ASC into THP-1 cells (blue) via F-actin positive TNT’s (red; Figure 3E,G,J). Interestingly, those cells that were pre-incubated with CytoB demonstrated a substantial reduction in mitochondrial transfer to THP-1 cells (Figure 3F,H,I). To further confirm TNT-mediated mitochondrial transfer, cocultures in the presence or absence of CytoB were assessed for the gene expression of CAP2, NEXN, and ACTN1 that are known to associate with F-actin synthesis. Whereas the expression of CAP2, NEXN, and ACTN1 increased by 1.5–45-fold (*p* < 0.001) in cocultures as compared to monocultures, CytoB treatment significantly decreased gene expression of all three markers (Figure 3K; *p* < 0.01). Taken together, the data suggest that mitochondrial transfer from iMSCs or ASCs to immune cells occurs via F-actin-positive tubular tunneling nanotubes.

### 3.4. MSC Suppresses T Cell Population

**Figure 4:**
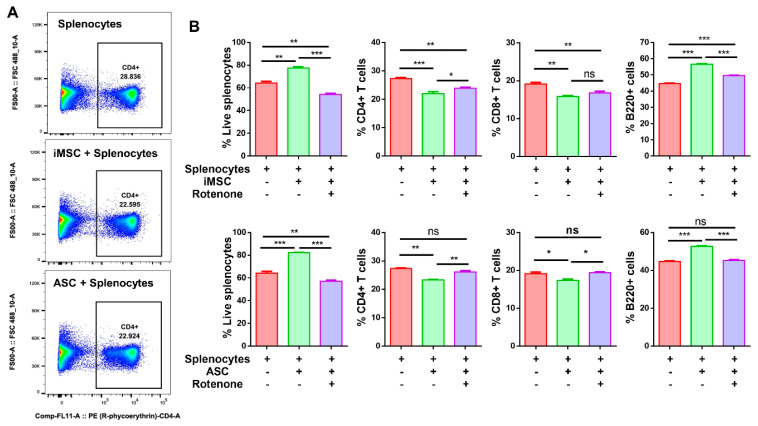
Both iMSC and ASC suppress effector T cell population. (**A**) Representative flow cytometry dot plots showing decreased CD4+ T cells in iMSC-splenocytes and ASC-splenocytes coculture compared to monoculture. (**B**) Quantification data represented as bar graphs after flow cytometry analysis of live cells, CD4+ T cells, CD8+ T cells, and B220+ cells in mouse splenocytes monocultures and cocultures with iMSC, ASC, as well as iMSC, and ASC pretreated with rotenone (500 nm, 2 h) at 1:10 (iMSC/ASC and splenocytes) ratio. Data shown as mean ± SEM from a single experiment repeated independently with similar results (* *p* < 0.01, ** *p* < 0.001, *** *p* < 0.0001 one-way ANOVA).

Different T cell populations of splenocytes interact closely; their ratio at a given time results from a balance between their mutual effects. Mitochondrial transfer to recipient cells can increase cell metabolism, resulting in cell division or cell differentiation. To better understand the impact of mitochondrial transfer to immune cells, we first assessed cell viability in mouse splenocyte coculture with either iMSC or ASCs. While the viability of splenocytes in monoculture is 48.7%, the viability in cocultures with iMSC and ASC increased to 65.7 and 64.3%, respectively (Figure 4B; *p* < 0.001). Similarly, the frequency of B220+ B lymphocyte cells in splenocytes cocultures with iMSC and ASC also significantly increased to 64.3 (*p* < 0.001) and 58.5%, respectively (Figure 4B; *p* < 0.01). Next, to better understand the effects of mitochondria transfer from stem cells to immune cells on their subpopulation, we assessed non-adherent splenocytes from cocultures with monoclonal antibodies for CD4, CD8, and B220 and compared them to monoculture in the presence or absence of rotenone. While the helper CD4+ T cells of splenocytes in monoculture is 21%, the coculture levels with iMSC and ASC significantly reduced to 15.4 and 15.9%, respectively (Figure 4B; *p* < 0.001). Similarly, 17% cytotoxic CD8+ T cells in splenocytes monoculture reduced to 12.9 and 12.8% in iMSC and ASC coculture with splenocytes, respectively (Figure 4B; *p* < 0.001). Interestingly, rotenone challenged cocultures demonstrated a small but significant increase in percent immune cells in both iMSC and ASC as compared to cells without rotenone. Altogether, these results indicate that both iMSCs and ASCs suppress the effector T cell population upon transferring their mitochondria to immune cells.

### 3.5. MSC Differentiates T Cells into Tregs and Suppresses CD69 Expression

**Figure 5:**
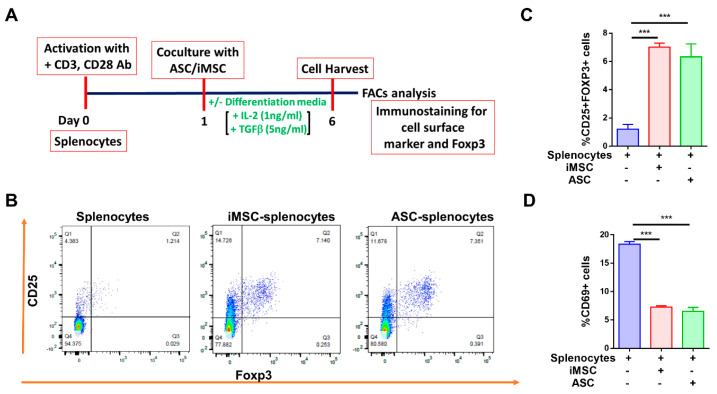
Both iMSC and ASC increase differentiation of T cells into Tregs and suppress CD69 expression. (**A**) Timeline and experimental details of iMSC/ASC coculture with mouse splenocytes (**B**) Representative flow cytometry dot plots showing increased CD25+Foxp3+ Tregs in iMSC/ASC cocultures with activated splenocytes. (**C**) Quantification of a percent increase in CD25+Foxp3+ cells with (**D**) a reduction in CD69+ cells in cocultures. Data shown as mean ± SEM from a single experiment repeated independently with similar results (*** *p* < 0.0001 one-way ANOVA).

The suppression of immune cell activation is one of the manifestations of MSC-mediated immunomodulation [12,26]. To assess whether mitochondrial transfer from stem cells to primary mouse splenocytes impacts T-cell differentiation, activated splenocytes were cocultured with iMSCs or ASCs and subsequently assessed for the expression of T regulatory cells (Figure 5A and Supplemental Figure S4). The dot plots from flow cytometry analysis clearly showed double-positive CD25+Foxp3+ cells in the upper right quadrant that were gated on CD4+ T cells (Figure 5B). While the level of CD25+Foxp3+ cells in monoculture is 2.7%, the levels in cocultures with iMSC and ASC increased to 7.1 and 7.4%, respectively (Figure 5B,C; *p* < 0.001). To further confirm the immunosuppression capability of iMSCs and ASCs, the expression of CD69, a potent immune activation marker, was evaluated. While the level of CD69+ cells in monoculture is 18.43%, the levels in cocultures with iMSC and ASC significantly decreased to 7.2 and 6.5%, respectively (Figure 5D; *p* < 0.001). Taken together, our data suggests that both iMSC and ASC manifest their immunomodulation via increased Treg population with a significant reduction in CD69+ cells in splenocyte coculture.

### 3.6. iMSC Significantly Increases Regulatory T Cells in the Retina of I/R Injured Mice

**Figure 6:**
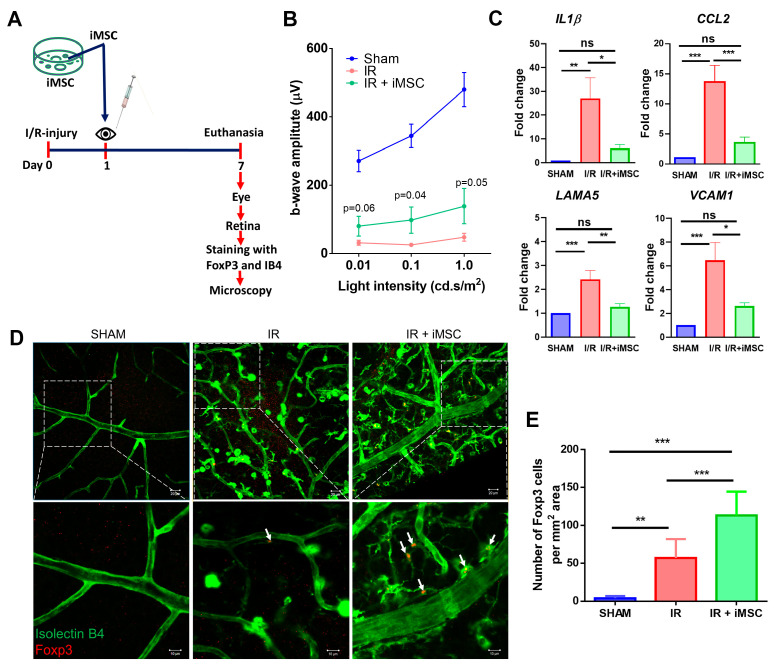
iMSC improves b-wave amplitudes, reduces inflammation correlated with increased regulatory T cells in retina of I/R injured mice. (**A**) Schematic representation of in-vivo experimental timeline and analyses. (**B**) B-wave amplitudes as measured by ERG show an expected decrease in I/R with a significant improvement in iMSC group. Data are shown as mean ± SEM, *n* = 7–13/group. *t*-test. (**C**) An increase in proinflammatory markers in I/R decreased with iMSC. Data are shown as mean ± SEM, one-way ANOVA. (**D**) Immunofluorescence images of Sham, I/R injured, and I/R injured with iMSC stained with isolectin B4 (green) and anti-Foxp3 (red) at day 7 post-injury. White arrows indicate Foxp3 cells. Boxed areas are shown at higher magnification below. (**E**) Quantification of Foxp3 cells significantly increased in I/R injured animals with iMSC compared to both Sham and I/R. Data shown as mean ± SEM, one-way ANOVA with Bonferroni correction (* *p* < 0.01, ** *p* < 0.001, *** *p* < 0.0001, ns = not significant).

MSC are well known to protect against retinal I/R damage [27,28,29,30]. To better understand if iMSC also can protect against retinal damage, we tested the intravitreal injection of iMSCs in the retinal I/R injury model in-vivo (Figure 6A). After 7 days, the retinal function, assessed by Electroretinogram (ERG), demonstrated improved b-wave amplitudes in I/R mice receiving iMSC as compared to saline-injected I/R eyes (at 1cd.s.m2 139 ± 48 v/s 48 ± 10 µvolt, *p* = 0.05) (Figure 6B). To further correlate the improved visual function observed with iMSC in I/R mice, gene expression analysis of proinflammatory markers was performed. Figure 6D shows normalized data of individual genes in all 3 groups of mice. I/R mice receiving saline had a significantly increased abundance of gene transcripts involved in microglial activation (IL1β) [31], endothelial activation (VCAM1, CCL2) [31,32], and T-cell regulation (LAMA5) [33] compared to sham mice. Interestingly, I/R mice receiving iMSC significantly ameliorated the increased gene expression (Figure 6C). The normalized fold change in expression of IL1β (I/R, 27.08 ± 8.69 vs. I/R+iMSC, 8.69 ± 1.54, *p* < 0.01), CCL2 (I/R, 13.63 ± 2.7 vs. I/R+iMSC, 3.52 ± 0.96, *p* < 0.0001), LAMA5 (I/R, 2.4 ± 0.37 vs. I/R+iMSC, 1.09 ± 0.09, *p* < 0.001) and VCAM1 (I/R, 7.4 ± 1.48 vs. I/R+iMSC, 2.2 ± 0.96, *p* < 0.01).

Subsequently, to correlate the iMSC ability to provide immunomodulation through increased Tregs, retinal flat mounts analyzed for Tregs in the retina by confocal microscopy revealed positive immunostaining (red) only in I/R and I/R+iMSC groups (Figure 6D). Neither the uninjured contralateral eye nor the iMSC injected into the uninjured eye was positive for Foxp3 expression (Supplemental Figure S5), suggesting the specificity of immunostaining and the correlation of Foxp3 upregulation to I/R injury. While the number of positive Foxp3 cells in the Sham group was 3.6 ± 1.2, the I/R injury retina demonstrated a significant upregulation with 57 ± 8.7 Tregs per mm2 area (*p* < 0.001). Interestingly, those I/R injury animals that received iMSC demonstrated a further significant increase in Tregs to 112 ± 11 cells per mm2 area (*p* < 0.0001; Figure 6E).

## 4. Discussion

Our study is the first to demonstrate that iMSC reprograms mouse CD4-T cells into Foxp3 regulatory Tregs to the best of our knowledge. Additionally, we show that intravitreal delivery of iMSCs in a retinal ischemia-reperfusion (I/R) injury model display therapeutic potential and suggests the mechanism of action may involve recruitment of Foxp3Tregs. Our in-vitro culture data show that iMSCs transferred mitochondria to immune T cells via F-actin nanotubes, suppressed effector T cells, and promoted differentiation CD4+CD25+Foxp3+ Tregs in coculture with mouse splenocytes on par with ASC. Importantly, we also demonstrated that the increased recruitment of Foxp3+Tregs in the retina correlated with dampened retinal inflammation and improved b-wave amplitudes in the I/R injury model. Our findings are in keeping with published data by others that have demonstrated Tregs act as part of the adaptive immune system, and thereby (A) serve as important regulators of inflammation and play a critical role in immune homeostasis [14]; (B) are inversely correlated with retinal ischemia [34]; and (C) are well known for immunomodulation, with recent evidence suggesting that MSCs can enhance Foxp3+Treg differentiation and stability from activated T cells in part through mitochondrial transfer [12,13,35].

Recent evidence suggests that MSCs can modulate Tregs [12], particularly Tregs expressing the Foxp3 transcription factor, part of the adaptive immune system and principal regulators of inflammation and immune homeostasis [14]. Although Tregs primarily originate in the thymus (tTregs), during tissue damage, the CD4+ (T_H_) cells at the site of injury can also be reprogrammed and acquire the Treg phenotype, also known as peripheral Tregs (pTregs) and regulate immunotolerance [19]. Vice-versa tTregs acquire CD4+ (T_H_) cell phenotype and become defective to perform its immunomodulatory function [35]. Thus, the ability of Tregs to migrate to the damaged tissue and dampen inflammation is an attractive strategy to curtail the ongoing inflammation [15,16,17]. Following this, we show increased Foxp3 positive cells in the retina after retinal damage that further increased with intravitreal injection of iMSC in the ischemic retina. This initial increase in Tregs under acute ischemia compared to sham injury aligns with a previous observation in the oxygen-induced retinopathy mouse model [28]; however, Treg numbers are likely insufficient to repair the retinal damage. On the other hand, those animals that received iMSC probably reached the optimal levels of Foxp3 cells to dampen the retinal damage. One limitation of our initial study is that it is unclear if the increased Foxp3 Tregs are reprogrammed from the local CD4+ T cells that were shown to be upregulated and causally linked to retinal damage [19] or were recruited from elsewhere. Another limitation is that how the increased Foxp3 Tregs regulate retinal tissue inflammation is not explored. Future studies beyond the scope of this study need further analysis on how iMSC induces Tregs and, thus, regulates retinal tissue damage.

Understanding the molecular basis of Treg generation and its stability is an active area of research, with several agents proposed to upregulate and stabilize Foxp3 expression [36,37]. Notably, some agents have even been shown to be effective under hypoxic conditions [38]. MSCs grown as “feeder cells” with Tregs significantly increased Treg cell number, suppressive function, and ex vivo expansion, primarily through mitochondrial transfer from adjacent MSCs, coupled with the promotion of Foxp3 cotranscriptional proteins [13]. Thus, a primary mechanism of action of MSCs may involve an increase in Treg cell number and/or function via Foxp3 stability. To this end, we show increased differentiation of Foxp3 cells from a mixed population of mouse splenocytes cocultured with iMSC, a feature also observed with ASC cocultured with whole human peripheral blood mononuclear cells [12]. One limitation of our current study is that it is unclear if the reprogramming of Foxp3 Tregs primarily occurs through mitochondrial transfer from adjacent iMSCs or other unknown mechanism(s). To this end, genome-wide patterns of DNA methylation of Foxp3 locus were implicated with FOXP3 gene expression, which determines the tTreg generation, pTreg generation, Treg stability, and tTreg and pTreg proliferation [39]. One possibility is that iMSC affects the epigenetic stability of the FOXP3 gene in Tregs, thus might increase its stability. Future studies need to explore these hypotheses.

MSCs help in the intercellular exchange of mitochondria to restore and regenerate the damaged tissue [9], specifically in various ocular cells [10]. Therefore, iMSC by mitochondrial transfer to Tregs or immune cells may modulate them towards more stable and functional pTregs phenotype in-vivo and aid in the suppressive ability to protect from I/R-injury. To this end, we show that iMSCs can transfer mitochondria to CD4+ T-cells and suppress the CD4+ cell population. Interestingly, both these activities are dependent on mitochondria transfer as evidenced by either blocking mitochondrial function via rotenone or the use of Cytochalasin-B, a known agent that blocks actin polymerization that caused a significant reduction in tunneling nanotubes and a significant reduction in mitochondria transfer to recipient cells. In support of our study, a previous study showed such tunnel nanotube-mediated transfer of mitochondria occurs in MSC [13]. The predominant mechanism of cytochalasin B is the inhibition of actin filament polymerization by binding to the end of growing filaments. Since F-actin filaments are dynamic in nature and their formation between donor and recipient cells is influenced by the activity of many actin-binding genes/proteins, we studied the transcriptional regulation of F-actin genes as also shown previously by Jiang et al. [10]. Based on our gene expression data, it is conceivable that cytochalasin B might indirectly affect transcriptional regulation of the F-actin-related genes and thus influences the mitochondrial transfer. Future studies are required to decipher the causal link of these actin-related genes to mitochondria transfer in our studies as mitochondria from MSCs are transferred to other cells via their exosomes [40] or gap junctions [41,42] or simply by non-mitochondrial paracrine factors [43]. Finally, one alternate hypothesis could be that a direct transfer of mitochondria, a phenomenon known as mitoception, shown for bone-marrow MSC, [44] should be explored for iMSC.

iMSC obtained in the current study were obtained by reprogramming human urine-derived epithelial cells with mRNA reprogramming, the fastest and most reliable reprogramming method to date [45]. Furthermore, our study demonstrates an iPSC line that has unlimited proliferation potential [21] and has the ease of obtaining without any surgical interventions will likely address the current challenges of cell therapies in the ischemic retina [46]. Furthermore, since iPSC lines can also be obtained from diseased or aged individuals [47], our studies will likely benefit future personalized medicine studies. In conclusion, our study demonstrates that iPSC-derived MSCs can transfer mitochondria to T cells to enhance differentiation into Foxp3 Tregs. Additionally, our current data indicate that MSC can improve the retina’s immune function by upregulation of Tregs to decrease inflammation and reduce I/R injury-induced retinal degeneration.

## Figures and Tables

**Table 1:** List of fluorophores-coupled FC antibody.

Antibody	Catalog Number	Manufacturer
CD4 (RM4-5)-PE	12-0042-82	eBioscience, San Diego, USA
CD8a (53-6.7)-PerCp/Cyanine 5.5	100733	Bio-legend, San Diego, USA
B220 (RA3-6B2)-AF700	103231	Bio-legend, San Diego, USA
CD69 (H1.2F3)-FITC	11-0691-82	eBioscience, San Diego, USA
CD25 (PC61)-V450	561257	BD Biosciences, Franklin Lakes, USA
Foxp3 (FJK-16s)-APC	17-5773-82	eBioscience, San Diego, USA

**Table 2:** List of Primer Sequences Used for SYBR green-based qPCR.

Gene	Forward	Reverse
*ACTN1*	5′-ACATGCAGCCAGAAGAGGAC-3′	5′-ACACCATGCCGTGAATGTCT-3′
*NEXN*	5′-ACGGAGGAGGAACGAAAACG-3′	5′-TGTCCTCAATCTGTTCAGCCC-3′
*CAP2*	5′-AGCTGTGTCTCCCAAACCTG-3′	5′-ACCCAATCCACATGACGCAA-3′
*18S*	5′-GCAATTATTCCCCATGAACG-3′	5′-GGCCTCACTAAACCATCCAA-3′

**Table 3:** List of Taqman assay IDs for qPCR.

Gene	Assay ID	Reference
18S ribosomal RNA (*18S*)	Mm04277571	NR_003278.3
Laminin, alpha 5 (*LAMA5*)	Mm01222029	NM_001081171.2
Chemokine (C–C motif) ligand 2 (*CCL2*)	Mm00441242	NM_011333.3
Vascular cell adhesion molecule 1 (*VCAM-1*)	Mm01320973_m1	NM_011693.3
Interleukin 1 β (*IL1β*)	Mm00434228_m1	NM_008361.3

## Data Availability

Data are available from the authors upon request.

## Supplementary Materials

The following are available online at https://www.mdpi.com/article/10.3390/cells10113006/s1, Figure S1: Gating strategy for iMSC characterization and representative plots. Overlay plots showing percent expression of MSC characteristic positive and negative surface markers on iMSC.; Figure S2: Flow cytometry representative plots showing splenocytes incubated with media obtained from MitoTrackerGreen stained ASC were negative for MitoTrackerGreen. ASC were stained with MitoTrackerGreen and cultured for 2 h. The cell supernatant was then incubated with splenocytes for 24 h, washed and assessed for MitoTrackerGreen.; Figure S3: Gatingstrategy for flow cytometry analysis and representative plots for transfer of mitochondria from iMSC to mouse T and B cells in vitro.; Figure S4: Representative gating strategy and flow cytometry dot plots showing CD25+Foxp3+ Tregs in iMSC/ASC co-cultures with activated splenocytes.; Figure S5: Retinal flat mounts analyzed for Tregs in the retin the iMSC injected contralateral eye (left) and un-injured cina by confocal microscopy revealed no posi-tive immunostaining ontralateral eye (right) for Foxp3 expression. IsolectinB4 staining was used to identify blood vessels. The data shown is representative of n = 3–4 eyes/group.

## References

[1] Park S.S. (2016). Cell Therapy Applications for Retinal Vascular Diseases: Diabetic Retinopathy and Retinal Vein Occlusion. Investig. Ophthalmol. Vis. Sci..

[2] Rivera J.C., Dabouz R., Noueihed B., Omri S., Tahiri H., Chemtob S. (2017). Ischemic Retinopathies: Oxidative Stress and Inflammation. Oxid. Med. Cell. Longev..

[3] Bertelli P.M., Pedrini E., Guduric-Fuchs J., Peixoto E., Pathak V., Stitt A.W., Medina R.J. (2020). Vascular Regeneration for Ischemic Retinopathies: Hope from Cell Therapies. Curr. Eye Res..

[4] Gaddam S., Periasamy R., Gangaraju R. (2019). Adult Stem Cell Therapeutics in Diabetic Retinopathy. Int. J. Mol. Sci..

[5] Rajashekhar G., Ramadan A., Abburi C., Callaghan B., Traktuev D.O., Evans-Molina C., Maturi R., Harris A., Kern T.S., March K.L. (2014). Regenerative therapeutic potential of adipose stromal cells in early stage diabetic retinopathy. PLoS ONE.

[6] Elshaer S.L., Evans W., Pentecost M., Lenin R., Periasamy R., Jha K.A., Alli S., Gentry J., Thomas S.M., Sohl N. (2018). Adipose stem cells and their paracrine factors are therapeutic for early retinal complications of diabetes in the Ins2(Akita) mouse. Stem Cell Res. Ther..

[7] Cerman E., Akkoc T., Eraslan M., Sahin O., Ozkara S., Vardar Aker F., Subasi C., Karaoz E., Akkoc T. (2016). Correction: Retinal Electrophysiological Effects of Intravitreal Bone Marrow Derived Mesenchymal Stem Cells in Streptozotocin Induced Diabetic Rats. PLoS ONE.

[8] Zhang W., Wang Y., Kong J., Dong M., Duan H., Chen S. (2017). Therapeutic efficacy of neural stem cells originating from umbilical cord-derived mesenchymal stem cells in diabetic retinopathy. Sci. Rep..

[9] Ahmad T., Mukherjee S., Pattnaik B., Kumar M., Singh S., Kumar M., Rehman R., Tiwari B.K., Jha K.A., Barhanpurkar A.P. (2014). Miro1 regulates intercellular mitochondrial transport & enhances mesenchymal stem cell rescue efficacy. EMBO J..

[10] Jiang D., Chen F.X., Zhou H., Lu Y.Y., Tan H., Yu S.J., Yuan J., Liu H., Meng W., Jin Z.B. (2020). Bioenergetic Crosstalk between Mesenchymal Stem Cells and various Ocular Cells through the intercellular trafficking of Mitochondria. Theranostics.

[11] Babenko V.A., Silachev D.N., Popkov V.A., Zorova L.D., Pevzner I.B., Plotnikov E.Y., Sukhikh G.T., Zorov D.B. (2018). Miro1 Enhances Mitochondria Transfer from Multipotent Mesenchymal Stem Cells (MMSC) to Neural Cells and Improves the Efficacy of Cell Recovery. Molecules.

[12] Fiori A., Uhlig S., Klüter H., Bieback K. (2021). Human Adipose Tissue-Derived Mesenchymal Stromal Cells Inhibit CD4+ T Cell Proliferation and Induce Regulatory T Cells as Well as CD127 Expression on CD4+CD25+ T Cells. Cells.

[13] Do J.S., Zwick D., Kenyon J.D., Zhong F., Askew D., Huang A.Y., Van’t Hof W., Finney M., Laughlin M.J. (2021). Mesenchymal stromal cell mitochondrial transfer to human induced T-regulatory cells mediates FOXP3 stability. Sci. Rep..

[14] Walker L.S. (2013). Treg and CTLA-4: Two intertwining pathways to immune tolerance. J. Autoimmun..

[15] Taams L.S., van Amelsfort J.M., Tiemessen M.M., Jacobs K.M., de Jong E.C., Akbar A.N., Bijlsma J.W., Lafeber F.P. (2005). Modulation of monocyte/macrophage function by human CD4+CD25+ regulatory T cells. Hum. Immunol..

[16] Maloy K.J., Salaun L., Cahill R., Dougan G., Saunders N.J., Powrie F. (2003). CD4+CD25+ T(R) cells suppress innate immune pathology through cytokine-dependent mechanisms. J. Exp. Med..

[17] Andre S., Tough D.F., Lacroix-Desmazes S., Kaveri S.V., Bayry J. (2009). Surveillance of antigen-presenting cells by CD4+ CD25+ regulatory T cells in autoimmunity: Immunopathogenesis and therapeutic implications. Am. J. Pathol..

[18] Khanh Vu T.H., Chen H., Pan L., Cho K.S., Doesburg D., Thee E.F., Wu N., Arlotti E., Jager M.J., Chen D.F. (2020). CD4(+) T-Cell Responses Mediate Progressive Neurodegeneration in Experimental Ischemic Retinopathy. Am. J. Pathol..

[19] Josefowicz S.Z., Lu L.F., Rudensky A.Y. (2012). Regulatory T cells: Mechanisms of differentiation and function. Annu. Rev. Immunol..

[20] Rajasingh S., Thangavel J., Czirok A., Samanta S., Roby K.F., Dawn B., Rajasingh J. (2015). Generation of Functional Cardiomyocytes from Efficiently Generated Human iPSCs and a Novel Method of Measuring Contractility. PLoS ONE.

[21] Rajasingh S., Sigamani V., Selvam V., Gurusamy N., Kirankumar S., Vasanthan J., Rajasingh J. (2021). Comparative analysis of human induced pluripotent stem cell-derived mesenchymal stem cells and umbilical cord mesenchymal stem cells. J. Cell. Mol. Med..

[22] Periasamy R., Elshaer S.L., Gangaraju R. (2019). CD140b (PDGFRbeta) signaling in adipose-derived stem cells mediates angiogenic behavior of retinal endothelial cells. Regen. Eng. Transl. Med..

[23] Lim J.F., Berger H., Su I.H. (2016). Isolation and Activation of Murine Lymphocytes. J. Vis. Exp..

[24] Namwanje M., Bisunke B., Rousselle T.V., Lamanilao G.G., Sunder V.S., Patterson E.C., Kuscu C., Kuscu C., Maluf D., Kiran M. (2021). Rapamycin Alternatively Modifies Mitochondrial Dynamics in Dendritic Cells to Reduce Kidney Ischemic Reperfusion Injury. Int. J. Mol. Sci..

[25] Jha K.A., Pentecost M., Lenin R., Gentry J., Klaic L., Del Mar N., Reiner A., Yang C.H., Pfeffer L.M., Sohl N. (2019). TSG-6 in conditioned media from adipose mesenchymal stem cells protects against visual deficits in mild traumatic brain injury model through neurovascular modulation. Stem Cell Res. Ther..

[26] Kronsteiner B., Wolbank S., Peterbauer A., Hackl C., Redl H., van Griensven M., Gabriel C. (2011). Human mesenchymal stem cells from adipose tissue and amnion influence T-cells depending on stimulation method and presence of other immune cells. Stem Cells Dev..

[27] Roth S., Dreixler J.C., Mathew B., Balyasnikova I., Mann J.R., Boddapati V., Xue L., Lesniak M.S. (2016). Hypoxic-Preconditioned Bone Marrow Stem Cell Medium Significantly Improves Outcome After Retinal Ischemia in Rats. Investig. Ophthalmol. Vis. Sci..

[28] Noueihed B., Rivera J.C., Dabouz R., Abram P., Omri S., Lahaie I., Chemtob S. (2021). Mesenchymal Stromal Cells Promote Retinal Vascular Repair by Modulating Sema3E and IL-17A in a Model of Ischemic Retinopathy. Front. Cell Dev. Biol..

[29] Mathew B., Ravindran S., Liu X., Torres L., Chennakesavalu M., Huang C.C., Feng L., Zelka R., Lopez J., Sharma M. (2019). Mesenchymal stem cell-derived extracellular vesicles and retinal ischemia-reperfusion. Biomaterials.

[30] Moisseiev E., Anderson J.D., Oltjen S., Goswami M., Zawadzki R.J., Nolta J.A., Park S.S. (2017). Protective Effect of Intravitreal Administration of Exosomes Derived from Mesenchymal Stem Cells on Retinal Ischemia. Curr. Eye Res..

[31] Gustavsson C., Agardh C.D., Hagert P., Agardh E. (2008). Inflammatory markers in nondiabetic and diabetic rat retinas exposed to ischemia followed by reperfusion. Retina.

[32] Wang L., Li C., Guo H., Kern T.S., Huang K., Zheng L. (2011). Curcumin inhibits neuronal and vascular degeneration in retina after ischemia and reperfusion injury. PLoS ONE.

[33] Zhang X., Wang Y., Song J., Gerwien H., Chuquisana O., Chashchina A., Denz C., Sorokin L. (2020). The endothelial basement membrane acts as a checkpoint for entry of pathogenic T cells into the brain. J. Exp. Med..

[34] Deliyanti D., Talia D.M., Zhu T., Maxwell M.J., Agrotis A., Jerome J.R., Hargreaves E.M., Gerondakis S., Hibbs M.L., Mackay F. (2017). Foxp3(+) Tregs are recruited to the retina to repair pathological angiogenesis. Nat. Commun..

[35] Zhou X., Bailey-Bucktrout S.L., Jeker L.T., Penaranda C., Martinez-Llordella M., Ashby M., Nakayama M., Rosenthal W., Bluestone J.A. (2009). Instability of the transcription factor Foxp3 leads to the generation of pathogenic memory T cells in vivo. Nat. Immunol..

[36] Colamatteo A., Carbone F., Bruzzaniti S., Galgani M., Fusco C., Maniscalco G.T., Di Rella F., de Candia P., De Rosa V. (2019). Molecular Mechanisms Controlling Foxp3 Expression in Health and Autoimmunity: From Epigenetic to Post-translational Regulation. Front. Immunol..

[37] Kanamori M., Nakatsukasa H., Okada M., Lu Q., Yoshimura A. (2016). Induced Regulatory T Cells: Their Development, Stability, and Applications. Trends Immunol..

[38] Someya K., Nakatsukasa H., Ito M., Kondo T., Tateda K.I., Akanuma T., Koya I., Sanosaka T., Kohyama J., Tsukada Y.I. (2017). Improvement of Foxp3 stability through CNS2 demethylation by TET enzyme induction and activation. Int. Immunol..

[39] Zheng Y., Josefowicz S., Chaudhry A., Peng X.P., Forbush K., Rudensky A.Y. (2010). Role of conserved non-coding DNA elements in the Foxp3 gene in regulatory T-cell fate. Nature.

[40] Monsel A., Zhu Y.G., Gennai S., Hao Q., Hu S., Rouby J.J., Rosenzwajg M., Matthay M.A., Lee J.W. (2015). Therapeutic Effects of Human Mesenchymal Stem Cell-derived Microvesicles in Severe Pneumonia in Mice. Am. J. Respir. Crit. Care Med..

[41] Islam M.N., Das S.R., Emin M.T., Wei M., Sun L., Westphalen K., Rowlands D.J., Quadri S.K., Bhattacharya S., Bhattacharya J. (2012). Mitochondrial transfer from bone-marrow-derived stromal cells to pulmonary alveoli protects against acute lung injury. Nat. Med..

[42] Li C., Cheung M.K.H., Han S., Zhang Z., Chen L., Chen J., Zeng H., Qiu J. (2019). Mesenchymal stem cells and their mitochondrial transfer: A double-edged sword. Biosci. Rep..

[43] Tasso R., Ilengo C., Quarto R., Cancedda R., Caspi R.R., Pennesi G. (2012). Mesenchymal Stem Cells Induce Functionally Active T-Regulatory Lymphocytes in a Paracrine Fashion and Ameliorate Experimental Autoimmune Uveitis. Investig. Ophthalmol. Vis. Sci..

[44] Court A.C., Le-Gatt A., Luz-Crawford P., Parra E., Aliaga-Tobar V., Bátiz L.F., Contreras R.A., Ortúzar M.I., Kurte M., Elizondo-Vega R. (2020). Mitochondrial transfer from MSCs to T cells induces Treg differentiation and restricts inflammatory response. EMBO Rep..

[45] Gaignerie A., Lefort N., Rousselle M., Forest-Choquet V., Flippe L., Francois-Campion V., Girardeau A., Caillaud A., Chariau C., Francheteau Q. (2018). Urine-derived cells provide a readily accessible cell type for feeder-free mRNA reprogramming. Sci. Rep..

[46] Bhattacharya S., Gangaraju R., Chaum E. (2017). Recent Advances in Retinal Stem Cell Therapy. Curr. Mol. Biol. Rep..

[47] Spitzhorn L.-S., Megges M., Wruck W., Rahman M.S., Otte J., Degistirici Ö., Meisel R., Sorg R.V., Oreffo R.O.C., Adjaye J. (2019). Human iPSC-derived MSCs (iMSCs) from aged individuals acquire a rejuvenation signature. Stem Cell Res. Ther..

