# Supplementary material for: Mesenchymal Stem Cell Induced Foxp3(+) Tregs Suppress Effector T Cells and Protect against Retinal Ischemic Injury
Source: Cells. 2021 Nov 4;10(11):3006. doi: 10.3390/cells10113006 (PMC8616393; doi:10.3390/cells10113006)
Supplement: Supplementary file 1 [file cells-10-03006-s001.zip › cells-1401542 - SM.pdf]

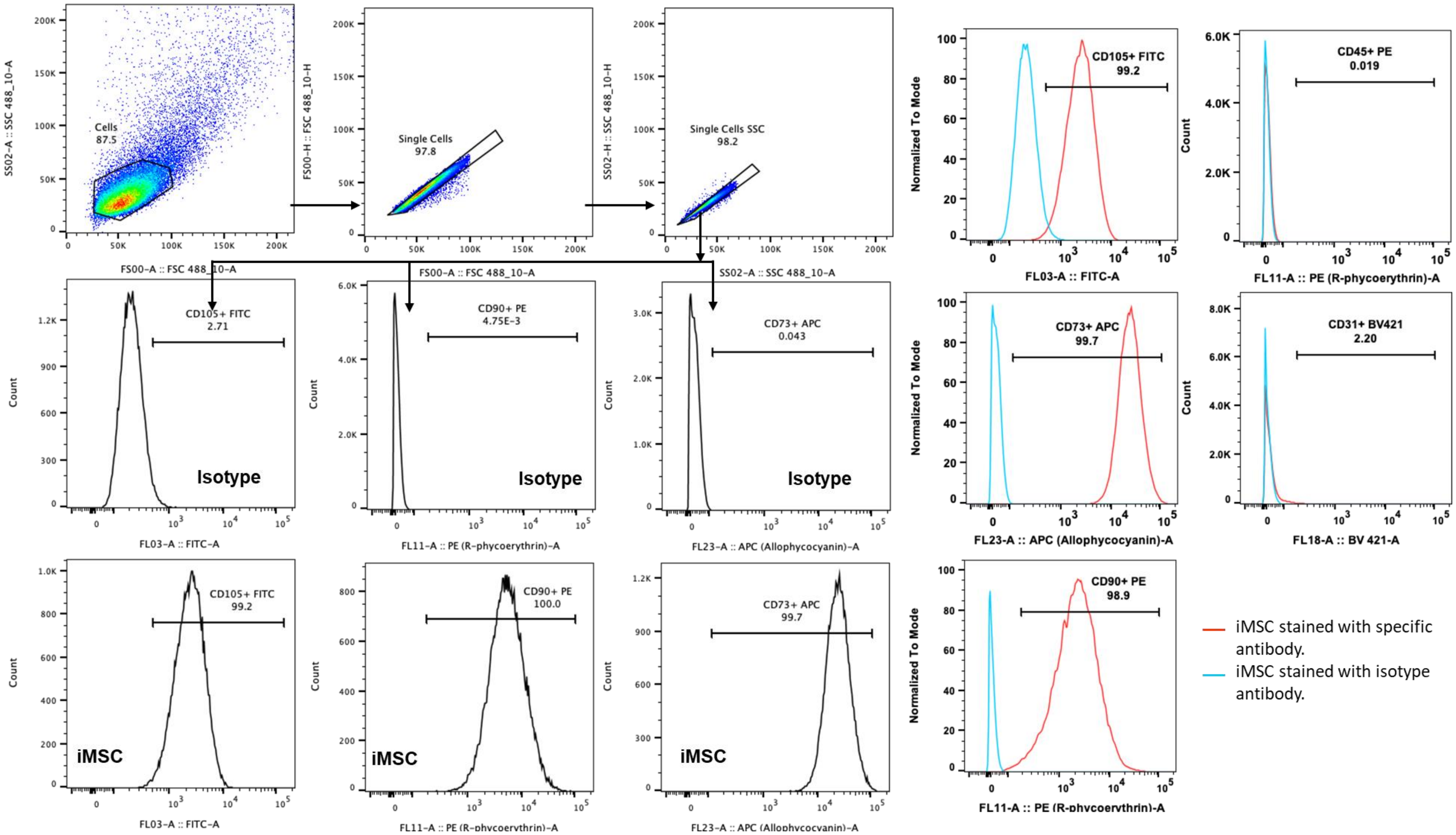

Supplementary Fig S1: Gating strategy for iMSC characterization and representative plots . Overlay plots showing MSC characteristics positive and negative surface markers percent expression on iMSC.

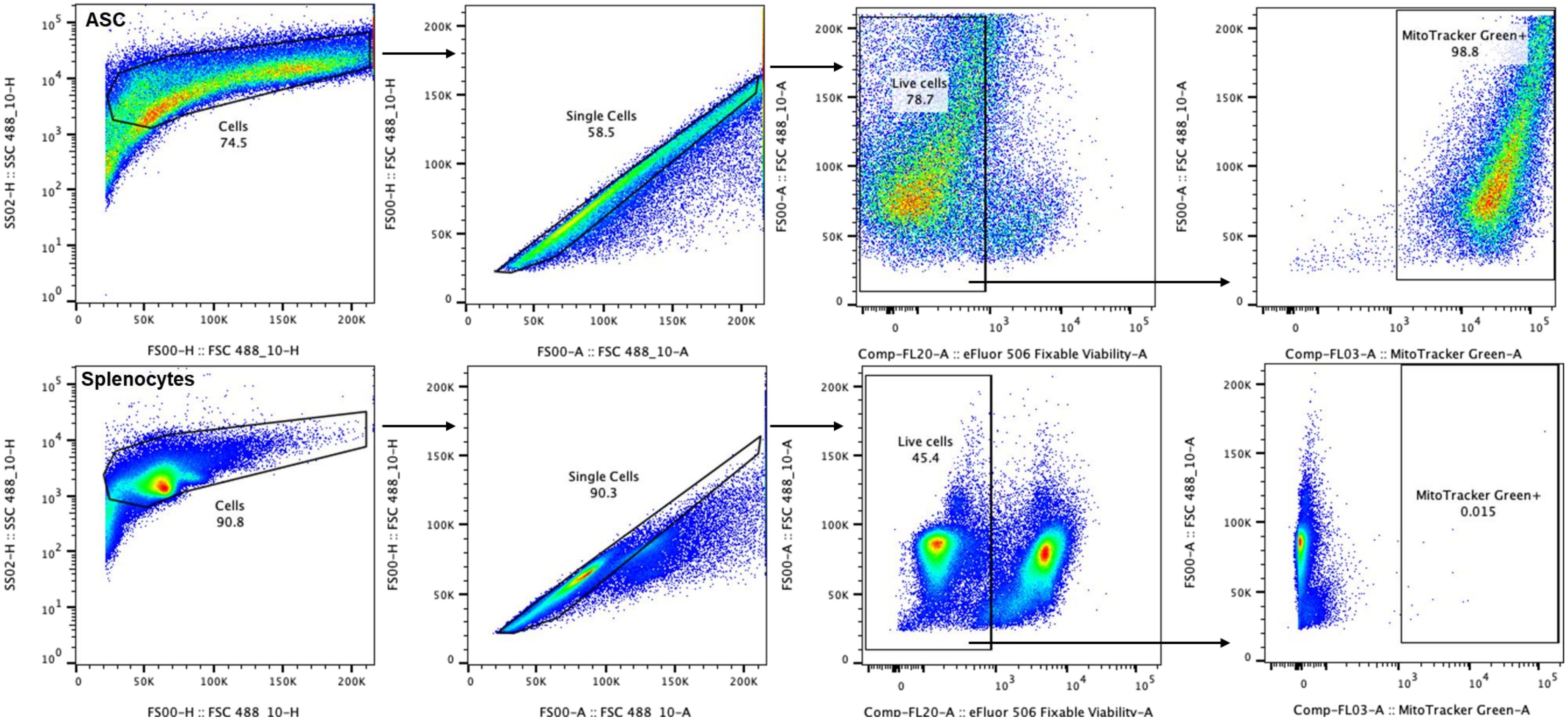

Supplementary Fig S2: Flow cytometry representative plots showing splenocytes incubated with media obtained from mitoTracker green stained ASC were negative for MitoTracker Green. ASC were stained with mitoTracker Green and cultured for 2 h. The cell supernatant was then incubated with splenocytes for 24h, washed and assessed for mitoTracker Green.

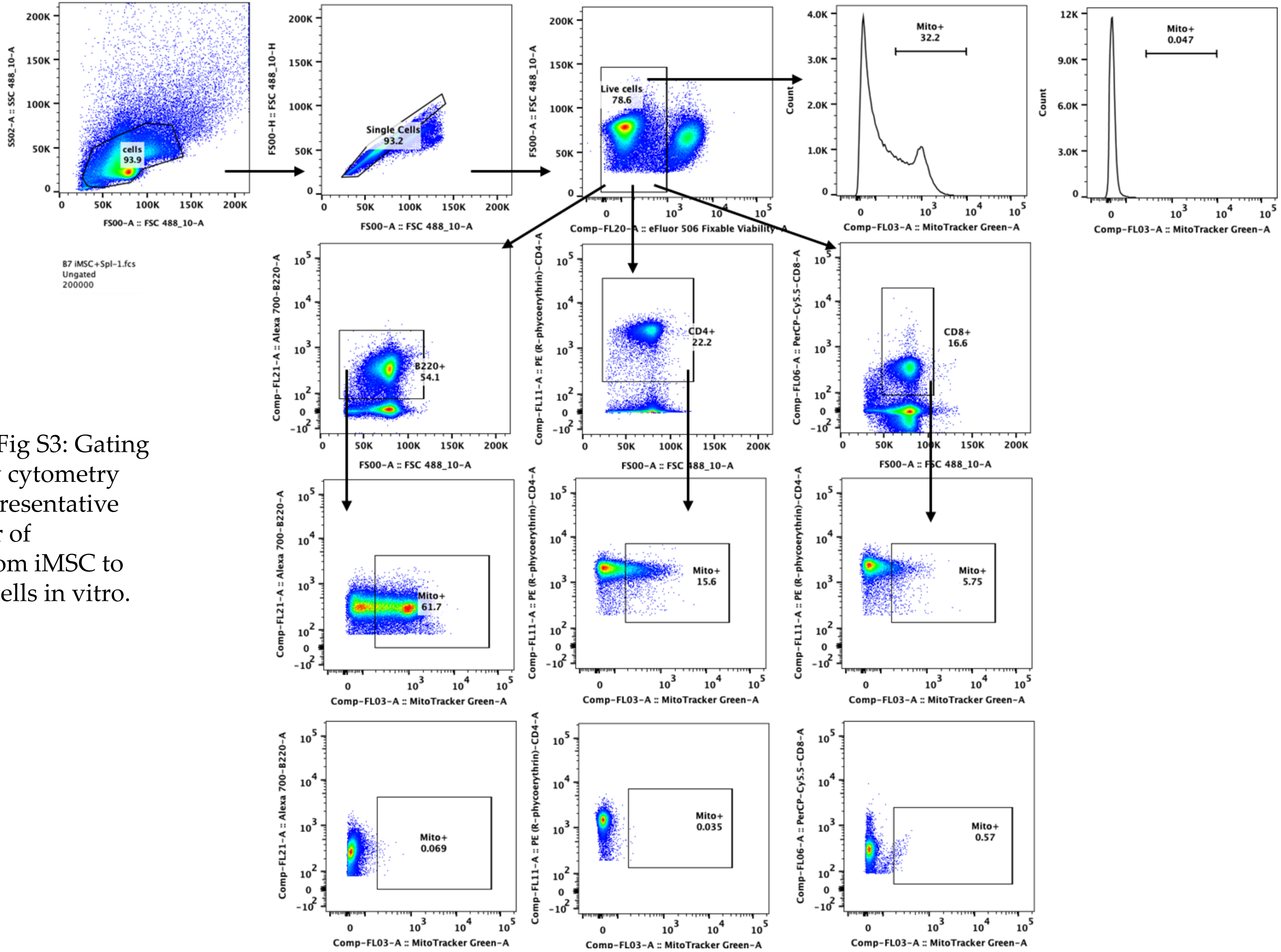

Supplementary Fig S3: Gating strategy for flow cytometry analysis and representative plots for transfer of mitochondria from iMSC to mouse T and B cells in vitro.

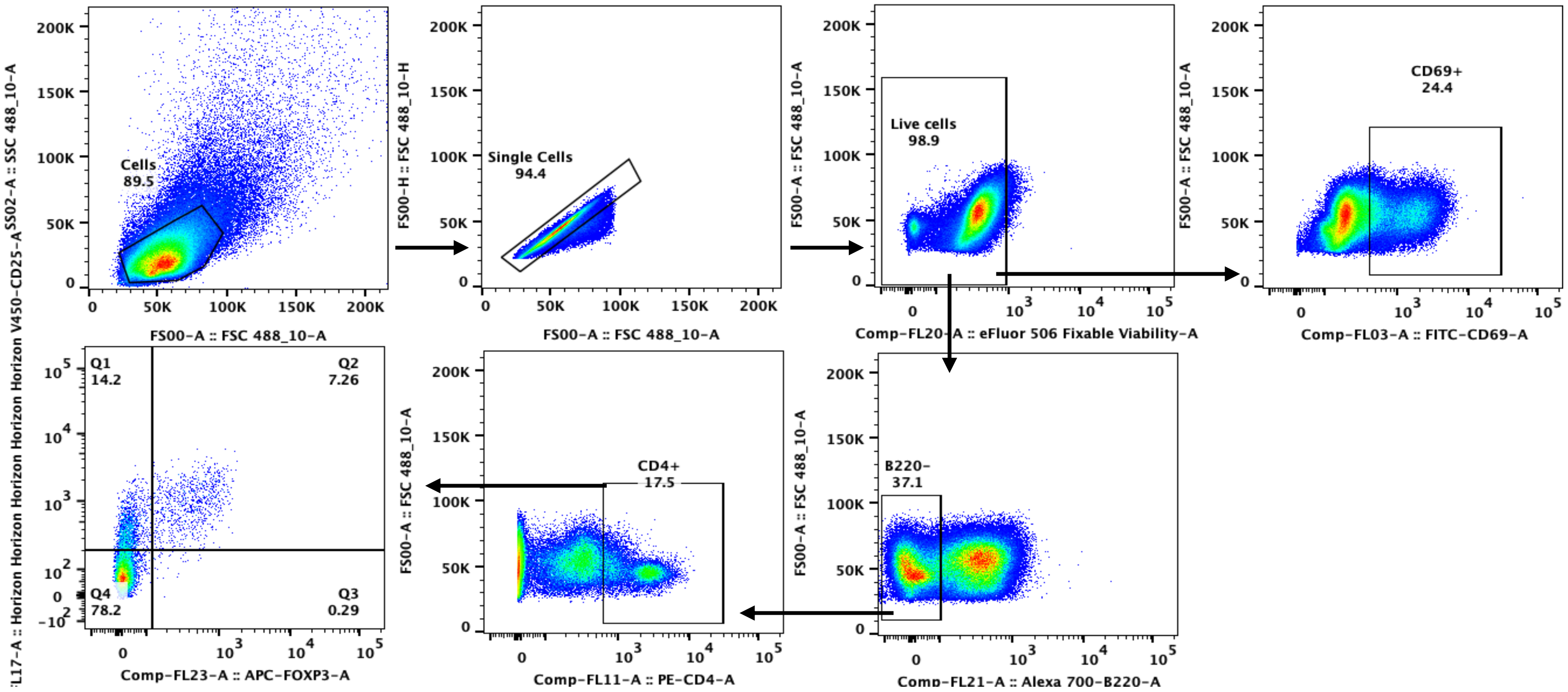

Supplementary Figure S4: Representative gating strategy and flow cytometry dot plots showing CD25+Foxp3+ Tregs in iMSC/ASC co-cultures with activated splenocytes.

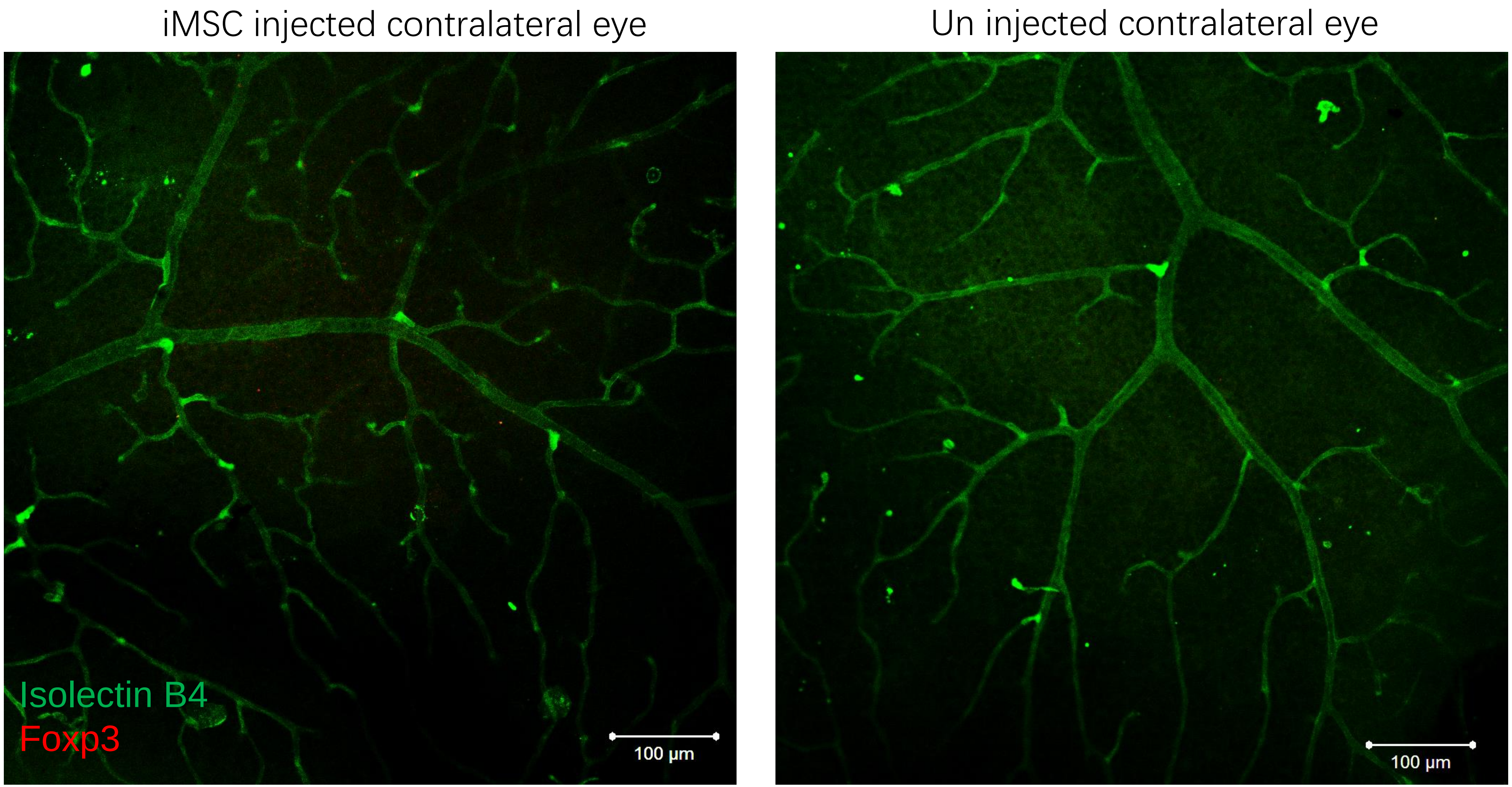

Supplementary Fig S5: Retinal flat mounts analyzed for Tregs in the retina by confocal microscopy revealed no positive immunostaining in the iMSC injected contralateral eye (left) and un-injured contralateral eye (right) for Foxp3 expression. Isolectin B4 staining was used to identify blood vessels. The data shown is representative of n=3-4 eyes/group.
